# Glycyrrhizin, an inhibitor of HMGB1 induces autolysosomal degradation function and inhibits* Helicobacter pylori* infection

**DOI:** 10.1186/s10020-023-00641-6

**Published:** 2023-04-10

**Authors:** Uzma Khan, Bipul Chandra Karmakar, Priyanka Basak, Sangita Paul, Animesh Gope, Deotima Sarkar, Asish Kumar Mukhopadhyay, Shanta Dutta, Sushmita Bhattacharya

**Affiliations:** 1 Division of Biochemistry ICMR-NICED, ICMR-National Institute of Cholera and Enteric Diseases (ICMR-NICED), Kolkata, 700010 India; 2 Division of Bacteriology ICMR-NICED, ICMR-National Institute of Cholera and Enteric Diseases (ICMR-NICED), Kolkata, 700010 India; 3 Division of Clinical Medicine, ICMR-NICED, ICMR- National Institute of Cholera and Enteric Diseases (ICMR-NICED), Kolkata, India

**Keywords:** *Helicobacter pylori*, Autophagy, Glycyrrhizin, HMGB1, LMP

## Abstract

**Background:**

*Helicobacter pylori* is a key agent for causing gastric complications linked with gastric disorders. In response to infection, host cells stimulate autophagy to maintain cellular homeostasis. However, *H. pylori* have evolved the ability to usurp the host’s autophagic machinery. High mobility group box1 (HMGB1), an alarmin molecule is a regulator of autophagy and its expression is augmented during infection and gastric cancer. Therefore, this study aims to explore the role of glycyrrhizin (a known inhibitor of HMGB1) in autophagy during *H. pylori* infection.

**Main methods:**

Human gastric cancer (AGS) cells were infected with the *H. pylori* SS1 strain and further treatment was done with glycyrrhizin. Western blot was used to examine the expression of autophagy proteins. Autophagy and lysosomal activity were monitored by fluorescence assays. A knockdown of HMGB1 was performed to verify the effect of glycyrrhizin. *H. pylori* infection in in vivo mice model was established and the effect of glycyrrhizin treatment was studied.

**Results:**

The autophagy-lysosomal pathway was impaired due to an increase in lysosomal membrane permeabilization during *H. pylori* infection in AGS cells. Subsequently, glycyrrhizin treatment restored the lysosomal membrane integrity. The recovered lysosomal function enhanced autolysosome formation and concomitantly attenuated the intracellular *H. pylori* growth by eliminating the pathogenic niche. Additionally, glycyrrhizin treatment inhibited inflammation and improved gastric tissue damage in mice.

**Conclusion:**

This study showed that inhibiting HMGB1 restored lysosomal activity to ameliorate *H. pylori* infection. It also demonstrated the potential of glycyrrhizin as an antibacterial agent to address the problem of antimicrobial resistance.

**Supplementary Information:**

The online version contains supplementary material available at 10.1186/s10020-023-00641-6.

## Background

Infection with *Helicobacter pylori* is one of the key factors responsible for causing gastric disorders and a major risk factor for progression to gastritis and gastric cancer. It is a gram-negative bacterium that has evolved with the ability to colonize and take refuge in epithelial cells of the stomach (Khatoon et al. 2016; Li et al. 2017; Jung et al. 2017; González et al. 2021). This is considered as one of the possible reasons owing to the rise of antibiotic resistance of *H. pylori* (Tshibangu-Kabamba et al. 2021; Thung et al. 2016). On account of this, WHO has considered *H. pylori* in the high-priority pathogens list (Shrivastava et al. 2018). Mounting evidence suggests that reprogramming host cellular pathways are an obligatory facet of *H. pylori* infection (Chmiela et al. 2017; Libânio et al. 2015Sierra et al. 2020). On the other end of the spectrum, to eliminate an incoming pathogen, the host often deploys several cellular defense strategies. Autophagy is one of the important pathways involved in recognizing and capturing intracellular bacteria for their degradation (Yang et al. 2016, 2018; Raju et al. 2012).* H. pylori* infection in epithelial cells often induces the host autophagic machinery during early infection while survival and colonization of *H. pylori* are favoured by inhibition of autophagy at later stages (Yang et al. 2018, 2022; Tang et al. 2012; Kim et al. 2018). *H. pylori* secreted effector proteins like CagA and VacA have an impact on autophagy during infection*.* CagA inhibits autophagy and helps in the survival of bacteria within the host (Terebiznik et al. 2009 and Tsugawa et al. 2019). Moreover, autophagy is dynamically altered in response to infection (Levine et al. 2011).

Prior studies have shown that High mobility group box 1 (HMGB1) is augmented during *H. pylori* infection (Lin et al. 2016). Research over the past has also established that HMGB1 induces pro-autophagic activities (Yin et al. 2017; Tang et al. 2010). However, the role of HMGB1-mediated autophagy in *H. pylori* infection is unknown. Keeping in mind this scenario of *H. pylori* infection and autophagy impairment; drug designing is inevitable as antibiotic resistance is well known. In this study, we have used an inhibitor of HMGB1, glycyrrhizin (Mollica et al. 2007) to explore the role of the autophagy-lysosomal pathway during *H. pylori* infection in both in vitro and in vivo conditions. Here, we observed that pharmacological inhibition of HMGB1 reduces *H. pylori* infection by inducing autophagosomal lysosomal maturation.

## Methods

### *Helicobacter pylori* culture

*Helicobacter pylori*, Sydney Strain SS1 (*cagA* + *, vacA* s2m2) were grown on brain heart infusion (BHI) agar (Difco, USA) containing 7% heat-inactivated horse serum (Invitrogen), antibiotics, and IsoVitaleX as mentioned previously (Saha et al. 2022). Plates were kept in a microaerophilic atmosphere at 37 °C for five to six days. Stock cultures were stored at − 70 °C for further usage. Isolates were re-streaked on fresh BHI agar and incubated for 24 h which was used for experimental studies. *H. pylori* resistant strain [OT-14(3)] (*cagA-, vacA* s2m2), (clarithromycin, metronidazole resistant) isolated from a gastric cancer patient at IPGMER and SSKM hospital, Kolkata was cultured with the same protocol.

### Cell culture

The human gastric cancer cell line AGS was gifted by Dr. Asish Kumar Mukhopadhyay (ICMR-NICED, Kolkata). AGS cells were grown in F12 media (Sigma-Aldrich) supplemented with 10% heat-inactivated FBS (Sigma, USA), 1% penicillin–streptomycin (Sigma, USA), and maintained in an incubator at 37 °C and 5% CO_2_.

### In vitro infection assay

A cell density of 0.5 × 10^6^ per 60 mm cell culture dish was plated. *H. pylori* SS1 culture was dissolved in sterile phosphate-buffered saline (PBS) and adjusted to an OD of 1 at 600 nm followed by centrifugation at 10,000 g for 10 min. The cells were starved overnight in 2 mL antibiotic and FBS- free incomplete F12 media. Cells were further infected with or without *H. pylori* with a multiplicity of infection (MOI) 1:100 for 4 h followed by gentamicin (100 μg/ml) treatment for 1 h, to kill the extracellular bacteria. Cells were then washed with PBS and incubated in fresh medium and treatment was done with glycyrrhizin GLZ (200 µM) for another 4 h. Cell lysis was performed by adding 0.1% saponin for 15 min at room temperature and serial dilution was prepared and then 100µL of diluted suspension were plated on BHIA plates to determine the number of invaded bacteria into the AGS cells. Colonies were then counted after 5–7 days of incubation. The CFU was determined by plating various serial dilutions of these bacterial suspensions on BHI agar plates (Hu et al. 2019). A similar assay was also performed for the *H. pylori*-resistant strain [OT-14(3)] with or without glycyrrhizin for 4 h. In the case of chloroquine and bafilomycin treatment, cells were infected with or without *H. pylori* with an (MOI) 1:100 for 4 h followed by gentamicin treatment for 1 h and further treatment was done with glycyrrhizin (200 µM) and/or chloroquine (50 μM) and/or bafilomycin (50 nM) for 4 h and/or 18 h.

### Real-time PCR

Further, cDNA was prepared from RNA utilizing a Thermo Scientific cDNA synthesis kit. SYBR green kit of Applied Biosystems was used for Quantitative PCR. ΔΔCt method was used to calculate and normalization was performed with the housekeeping gene control GAPDH. ΔΔCt = test − internal control-test control. The relative density of *H. pylori* was quantified by performing semi-quantitative PCR, detecting *H. pylori*- specific 16S-ribosomal DNA (rDNA) primer, FP (5ʹ-AGAGAAGCAATACTGTGAA- 3ʹ) & RP (5ʹ-CGATTACTAGCGATTCCA- 3ʹ). GAPDH was measured for normalization, FP (5′-GTCTTCACCACCATGGAGAAGGC-3′), and RP (5′-CATGCCAGTGAGCTTCCCGTTCA-3′). The PCR efficiency for both the test gene (93%) and GAPDH (96%) are within the desired efficiency range which is 90–105% (Kralik et al. 2017).

### Immunofluorescence

For immunofluorescence staining, cells were fixed in 4% paraformaldehyde at room temperature for 1 h and blocked in PBS containing 3% BSA and 0.01% Triton X100 for 1 h. Next, coverslips were incubated with anti- LAMP1 and anti-Galectin-3 at 4 °C overnight. Subsequently, secondary antibody incubation was done using TRITC-conjugated anti-rabbit secondary antibody (1:1000) (Cat# AP132R) and FITC- conjugated anti-mouse secondary antibody (1:1000). Lastly, the coverslips were mounted on glass slides by adding ProLong™ Gold Antifade reagent with DAPI (Thermo Fisher) and examined using an inverted confocal microscope (Carl Zeiss LSM 710). For LAMP1 and LC3B immunofluorescence staining, the same protocol was followed.

### Transfection of plasmid and siRNA

The tandem fluorescent LC3B (tfLC3B) plasmid was a gift from Dr. Dhiraj Kumar ICGEB, New Delhi, India. To examine autophagosomes and autolysosomes, AGS cells were transiently transfected with tf-LC3B plasmid using Lipofectamine 2000 (Invitrogen). 48 h after transfection, cells were incubated with or without *H. pylori* and treated with glycyrrhizin. In the end, coverslips were mounted on ProLong™ Gold Antifade reagent with DAPI (Thermo Fisher) and imaged using an inverted confocal microscope. The following siRNAs were purchased from IDT: ATG5siRNA (ID hs.Ri. ATG5.13.1) and HMGB1 siRNA (ID hs.Ri.HMGB1.13.1) and used for transfection at 70% confluence with siRNA/ Non-specific siRNA using lipofectamine 2000 in a 35 mm dish. 48 h after transfection, cells were infected with or without *H. pylori* as described.

### Live-cell confocal microscopy

AGS cells (2 × 10^5^) were seeded on coverslips. After 24 h, cells were incubated with *H. pylori* (MOI 100) for 4 h followed by drug treatment for 4 h.

#### GFP-LAMP1

To monitor the lysosomes expressing LAMP1, *H. pylori*-infected and drug-treated AGS cells were subjected to live-cell imaging by adding 5 µl of baculovirus expressing Lamp1-GFP construct (Cell Light™ Lysosomes-GFP, BacMam 2.0, #C10507) for 16 h. Finally, cells were observed in the confocal microscope.

#### LysoTracker staining

To investigate the acidification of lysosomes, cells were incubated with LysoTracker Red DND-99 (Invitrogen, L7528) for 30 min. Cells were then observed under an inverted confocal microscope.

#### Dextran staining

Lysosomal destabilization was examined using Dextran, Alexa Fluor™ 488, and 10,000 MW (D22910, Invitrogen). Cells were incubated with 200 µg/ml dextran for 2 h at 37 °C after infection and drug treatment was then observed under an inverted confocal microscope.

### MTT assay

Cellular toxicity was examined using a Colorimetric Cell Viability Kit (MTT) (Promokine) in 96-well plates. MTT reagent (3-(4,5-dimethylthiazol-2-yl)-2,5-diphenyltetrazolium bromide) was added and kept for 4 h. Purple crystal formazan formed was solubilized with DMSO. The amount of formazan salt was measured in a microplate reader (Bio-Rad Serial no. 19901) at an OD of 590 nm.

### Measurement of reactive oxygen species (ROS) levels

Intracellular ROS levels were monitored by using 2,7-dichlorodihydrofluorescein diacetate (DCFH-DA). 10 μM of DCFH-DA was added to control, infected, and drug-treated cells for 30 min and kept at 37 °C. Excess DCFH-DA was washed with PBS three times. Finally, fluorescence was monitored (Ex-485 nm and Em-520 nm) using a multimode reader, Molecular devices Spectramax M2.

### Immunoblotting

Control, drug-treated and infected cells were lysed in RIPA (Radio immunoprecipitation assay buffer) lysis buffer containing protease and phosphatase inhibitors. After cell lysis, centrifugation was done at 7000 rpm for 20 min at 4 °C. Further, protein level was determined and run on 10% or 12.5% SDS- PAGE gel at 120 V. Gels were further transferred to the PVDF membrane. 5% skimmed milk dissolved in TBST (20 mM Tris–HCl, 150 mM NaCl, 0.1% Tween20) buffer was used for blocking and incubated for 1 h at room temperature. In the next step, the membranes were kept overnight with primary antibodies at 4 °C. Eventually, after secondary antibody incubation, membranes were developed and scanned in a ChemiDoc. The primary antibodies used are rabbit polyclonal Anti-SQSTM1/P62 antibody (Cat# ab91526), rabbit monoclonal anti- ATG5 (Cat #ab228668), mouse monoclonal anti- LAMP1 (Cat# 15665S), rabbit monoclonal anti-beclin1 antibody (Cat #ab207612), rabbit polyclonal anti-LC3B antibody (Cat#ab51520), mouse polyclonal anti-β-actin (Cat #sc-47778), mouse monoclonal anti-Galectin-3 (Cat #sc-53127), rabbit polyclonal anti-α-Tubulin (Cat #BB-AB0118), anti-rabbit secondary HRP-conjugate (Cat #12-348), anti-mouse secondary HRP-conjugate (Cat #12-349).

### Enzyme-linked immunosorbent assay (ELISA)

Pro-inflammatory cytokine (IL-8, IL-6) levels from media and serum were estimated using the Krishgen Biosystems kit as per the manufacturer’s instructions. All experiments were done in triplicate.

### *H. pylori* infection in C57BL/6 mice and treatment with glycyrrhizin

Mice were maintained in the animal house under 12-h dark–light cycles. Experiments were conducted under the guidelines of the Institutional Animal Ethical Committee, NICED, Kolkata (PRO/157/- 260 July 2022). 8 weeks of male C57BL/6 mice bred in-house were used for the experiments. Three different experimental sets of mice were grouped: Control group (CON), n = 5, *H. pylori* SS1 infected group (HP), n = 5, infected group treated with GLZ (HP + GLZ), n = 5. All groups of mice were treated every day for seven days with an antibiotic cocktail (Ciprofloxacin, Metronidazole, Erythromycin, Albendazole) to avoid any other bacterial/ parasite infections. A Group of mice (HP & HP + GLZ) were inoculated with 10^8^ CFU/mouse/inoculation of *H. pylori* SS1 on three alternative days or PBS (CON). After two weeks of inoculation, a group of mice (HP + GLZ) was orally injected with glycyrrhizin (10 mg/kg) for 4 weeks, while a group of mice (CON) received sterile water. At the end of week 4, all mice were sacrificed. Gastric tissues were isolated and blood was collected for experimental purposes as described previously (Saha et al. 2022). Experiments were repeated three times. The total number of mice in each set of experiments was 15.

### Statistical analysis

All data were represented as mean ± S.E.M. Two groups were compared using an Unpaired t-test, and multiple comparisons were done by one-way ANOVA. The significance level has been marked as, * for p < 0.05, which implies significance, ** for p < 0.01, which implies very significance, and *** for p < 0.001, which implies highly significant.

## Results

### Glycyrrhizin induces autophagy in gastric epithelial cells

**Fig. 1:**
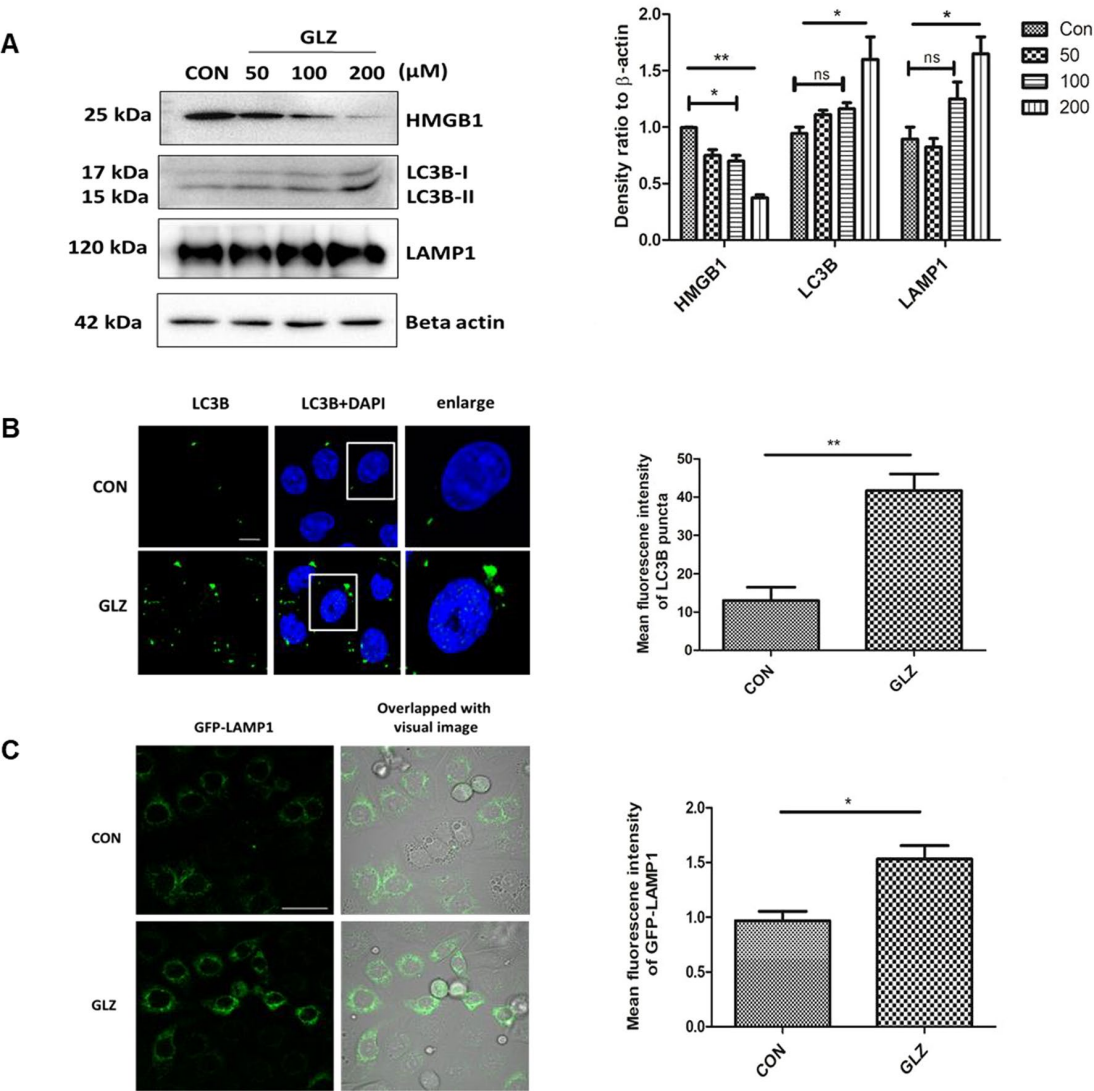
Glycyrrhizin treatment overexpresses autophagy proteins. AGS cells were treated with glycyrrhizin GLZ (200 µM) or DMSO for 4 h **a** Cell lysates were prepared and expression level of HMGB1 and autophagy marker proteins LC3B-II and LAMP1 was observed by western blot analysis. Beta-actin was used as a protein loading control. Densitometry analyses are represented graphically. **b** Control and drug-treated cells were subjected to immunofluorescence and changes in the mean fluorescence intensity were measured. Scale bar: 5 μm. Confocal microscopy showed LC3B puncta (green) formation. LC3B puncta formation was quantified and graphically plotted. **c** Live cell imaging was performed using a construct, LAMP1-GFP for labeling lysosomes under confocal microscopy. Fold change in the mean fluorescence intensity of GFP-LAMP1 was calculated. Scale bar: 10 μm. Graphs were represented as mean ± SEM (n = 3); Unpaired t-test was done and significance was calculated; *p < 0.05 and **p < 0.01

Previous reports indicated that glycyrrhizin induces autophagy in myoblast cells but there are no such reports in gastric cells till date (Lv et al. 2020). Here, we checked the expression levels of different autophagy proteins upon glycyrrhizin treatment in AGS gastric cancer cells. Glycyrrhizin treatment for 4 h elevated the expression of autophagy proteins LC3B-II and LAMP1 in a dose-dependent manner (100 µM, 200 µM). At 50 µM dose of glycyrrhizin, expression of LC3B-II and also LAMP1 was found to be almost the same. At 100 µM dose of glycyrrhizin treatment, although LC3B-II and LAMP1 increased but it was not statistically significant (p value for LC3B-II is 0.2334 and for LAMP1 is 0.3152). Interestingly, further increase in glycyrrhizin concentration e.g., at 200 µM dose, expression of LC3B-II increased significantly by 1.5 fold change (p value: 0.0136) and LAMP1 increased by 1.65 fold change (p value: 0.0419). On the other hand, glycyrrhizin reduced HMGB1 expression with an increase in concentration such as at 50 μM by 1.3 fold change (p value: 0.0332), at 100 μM by 1.4 fold change (p value: 0.0175) and at 200 μM by 2.4 fold change (p value: 0.0011) respectively (Fig. 1A). The most effective dose was 200 μM. To examine the possibility of toxicity of glycyrrhizin on AGS cells, we checked the effect of glycyrrhizin on the viability of AGS cells (Additional file 1: Fig. S1A). Glycyrrhizin treatment for 24 h at different concentrations (50, 100, 200 μM) did not show significant toxicity. Further, we confirmed glycyrrhizin-induced autophagy by immunofluorescence of LC3B. Drug treatment for 4 h showed a significant enhancement of LC3B puncta formation by 3.2 fold change (unpaired t-test and p value: 0.0071) (Fig. 1B). Subsequently, we assessed the effect of glycyrrhizin-induced autophagosomal maturation in gastric cancer cells. LAMP1 is known to be a marker for lysosomal activity, therefore, we labeled lysosomes with LAMP1-GFP construct after exposure to glycyrrhizin treatment. Subsequently, glycyrrhizin induced LAMP1 expression significantly by a factor of 1.587 (p value: 0.0191) in live cells as compared to control (Fig. 1C). Together, these results suggest that glycyrrhizin induces an autophagic response in gastric cells.

### Glycyrrhizin-induced autophagy inhibits intracellular *H. pylori* growth

**Fig. 2:**
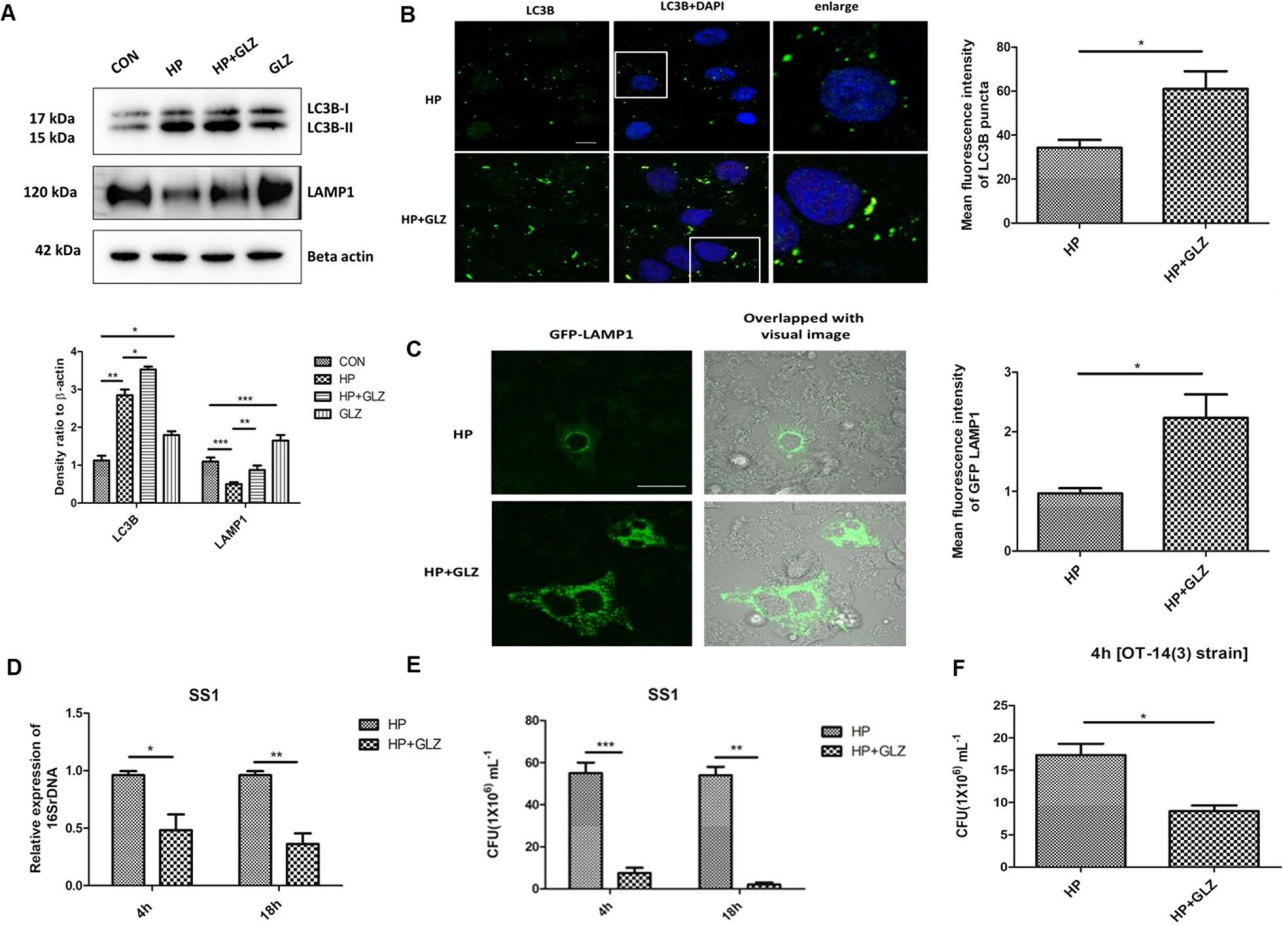
Exposure to Glycyrrhizin reduces intracellular *H. pylori* growth in AGS cells. **a–c** Infection with *H. pylori* SS1 strain (MOI 100) was performed in cells for 4 h and further exposed to glycyrrhizin (GLZ) (200 µM) for 4 h. **a** Immunoblotting was performed for quantification of autophagy-associated marker proteins (LC3B-II and LAMP1). Beta-actin was used as a loading control. Densitometry analyses are represented graphically. One-way ANOVA was performed. **b** Confocal microscopy showed LC3B puncta (green) in *H. pylori* (HP) infected & *H. pylori* + glycyrrhizin (HP + GLZ) treated cells, LC3B puncta formation was quantified and change in the mean fluorescence intensity was measured and graphically plotted. Scale bar: 5 μm. **c** Live cell imaging of LAMP1 under confocal microscopy showed GFP-LAMP1 puncta formation. Fold change in the mean fluorescence intensity of GFP-LAMP1 was calculated, unpaired t-test was performed and graphically represented Scale bar: 10 μm. **d, e** Cells were incubated with *H. pylori* SS1 strain (MOI 100) for 4 h followed by gentamicin treatment to kill extracellular bacteria. Finally, cells were treated with glycyrrhizin GLZ (200 µM) at two different time points for 4 h and 18 h **d** Intracellular *H. pylori* DNA (16SrDNA) was determined by real-time PCR. GAPDH was used as the internal control. **e** Cells were lysed and plated on BHIA plates with serial dilutions, for 4–5 days for counting colonies and CFU/ml was graphically represented. **f** Infection with *H. pylori* resistant strain [OT-14(3)] (MOI 100) for 4 h was performed in gastric cells followed by glycyrrhizin GLZ (200 µM) treatment for 4 h and CFU/ml was graphically represented. Graph were represented as mean ± SEM (n = 3); Unpaired t-test was done and significance was calculated; *p < 0.05, **p < 0.01 and ***p < 0.001

Since *H. pylori* is known to invade gastric epithelial cells, we examined the effect of drug treatment on the expression of autophagy proteins by immunoblotting in *H. pylori*-infected gastric cancer cells. *H. pylori* Sydney Strain SS1 was used for infection in gastric cells for 4 h and post-treatment was done with 200 µM glycyrrhizin (4 h). We observed upregulation of LC3B-II by 1.38 fold (p value: 0.0254) and LAMP1 by 1.74 (p value: 0.0028) in glycyrrhizin-treated *H. pylori*-infected cells as compared to the only *H. pylori*-infected cells (Fig. 2A). Moreover, LC3B-II by 1.63 fold (p value: 0.0123) and LAMP1 by 1.5 fold (p value: 0.0002) were also upregulated in glycyrrhizin-treated control cells as previously explained in (Fig. 1A, B). To verify the findings of LC3B and LAMP1 expression, we additionally performed an immunofluorescence assay and live-cell analysis of drug-treated and *H. pylori-*infected cells. Consistently, in comparison to untreated infected cells, glycyrrhizin treatment resulted in 2.5 fold (p value: 0.0252) increased LC3B puncta formation and 2.3 fold (p value: 0.0347) higher LAMP1 expression (Fig. 2B, C). The results showed that autophagosomal and lysosomal activities are increased by glycyrrhizin.

Next, we sought to examine the effect of autophagy induction on intracellular bacterial growth. We performed real-time PCR (RT-PCR) and checked *H. pylori*-specific 16SrDNA. Intracellular *H. pylori* level was significantly reduced by glycyrrhizin treatment for 4 h by 2.08 fold (p value: 0.0381) and 18 h by 2.7 fold (p value: 0.0037) (Fig. 2D). Of interest, we additionally examined the bacterial proliferation by bacterial adhesion assay. In line, intracellular *H. pylori* burden decreased significantly due to drug exposure for 4 h by tenfold (p value: 0.0005) and 18 h by 14.7 (p value: 0.0011) (Fig. 2E). Since antimicrobial resistance is a problem to curb *H. pylori* infection, we treated a resistant strain of *H. pylori* with glycyrrhizin for 4 h in AGS cells. Glycyrrhizin significantly reduced the growth of *H. pylori-*resistant strain [OT-14 (3)] by a factor of 2.0 (p value: 0.0117) (Fig. 2F). However, glycyrrhizin failed to reduce *H. pylori* growth in BHIA media which indicates glycyrrhizin has no direct bactericidal effect on *H. pylori* at 200 μM concentration (Additional file 1: Fig. S2A). Taken together, these data indicate that glycyrrhizin induces autophagy in gastric cancer cells and inhibits intracellular *H. pylori* growth.

### Enhancement of autophagic flux by glycyrrhizin contributed to anti-*H. pylori* activity

**Fig. 3:**
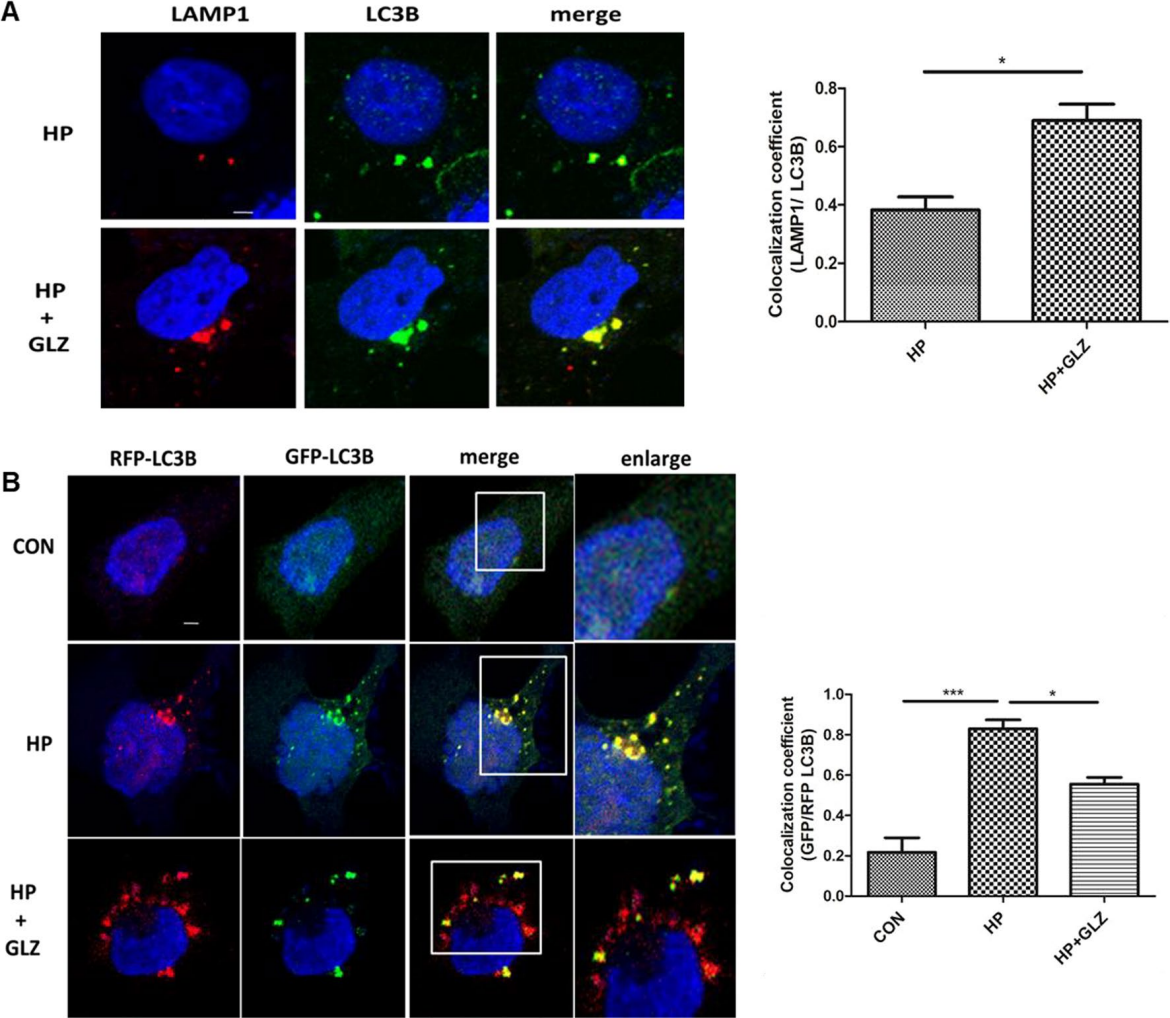
Activation of autophagic flux by glycyrrhizin treatment **(a, b)** Cells were incubated with *H. pylori* SS1 strain (4 h) followed by glycyrrhizin GLZ (200 µM) exposure for 4 h. **a** Glycyrrhizin (GLZ) exposure to *H. pylori*-infected cells for 4 h was subjected to LAMP1 and LC3B double immunofluorescence. Confocal microscopy showed yellow puncta with co-localization of LC3B puncta (green) & LAMP1 (red). Change in the co-localization coefficient of each group was calculated. Scale bar: 2 μm. **b** AGS cells were transfected with a tandem mRFP-GFP tag (tfLC3B) plasmid & further infected with *H. pylori* SS1 strain (MOI 100) for 4 h and finally, glycyrrhizin (GLZ) (200 µM) treatment was done for 4 h. Confocal microscopy showed LC3B puncta formation in control (CON), *H. pylori* (HP) infected & *H. pylori* + glycyrrhizin (HP + GLZ) treated cells. The yellow puncta showed autophagosomes. The free red puncta are autolysosomes. Change in the co-localization coefficient of each group was calculated. Scale bar: 2 μm. Graph represented as mean ± SEM (n = 3); Significance was determined by Unpaired t-test; *p < 0.05, ***p < 0.001

As *H. pylori* infection is involved in defective autophagosomal lysosomal maturation and degradation, we determined the effect of glycyrrhizin on autophagic flux. To assess the activation of autophagic flux by glycyrrhizin, we performed a double-immunofluorescence assay for both LC3B and LAMP1 protein. Results demonstrated that both LC3B and LAMP1 colocalized in *H. pylori*-infected and glycyrrhizin-treated infected cells (Fig. 3A). The data indicated that glycyrrhizin-induced autophagosomal lysosomal maturation by 1.8 fold (p value: 0.0125). Next, we examined the stage of glycyrrhizin-mediated autophagic degradation in both autophagosomes and lysosomes by transfecting the AGS cells with tandem fluorescent LC3B (tfLC3B) plasmid. In the case of *H. pylori*-infected gastric cells, autophagosomes appeared as yellow dots due to the colocalization of both GFP and RFP (fold change: 3.8 and p value: 0.0004). On the other hand, we observed more free red dots as GFP and RFP did not colocalize in infected cells followed by glycyrrhizin treatment (fold change: 1.5 and p value: 0.0238). Autolysosomes appear red due to the acidic pH of lysosomes which quench GFP (Fig. 3B). All together, these results revealed that glycyrrhizin prevents *H. pylori*-mediated inhibition of autolysosome formation in the infected cells by promoting lysosomal maturation.

### Anti- *H. pylori* effect and autophagic degradation by glycyrrhizin is mediated through HMGB1 inhibition

**Fig. 4:**
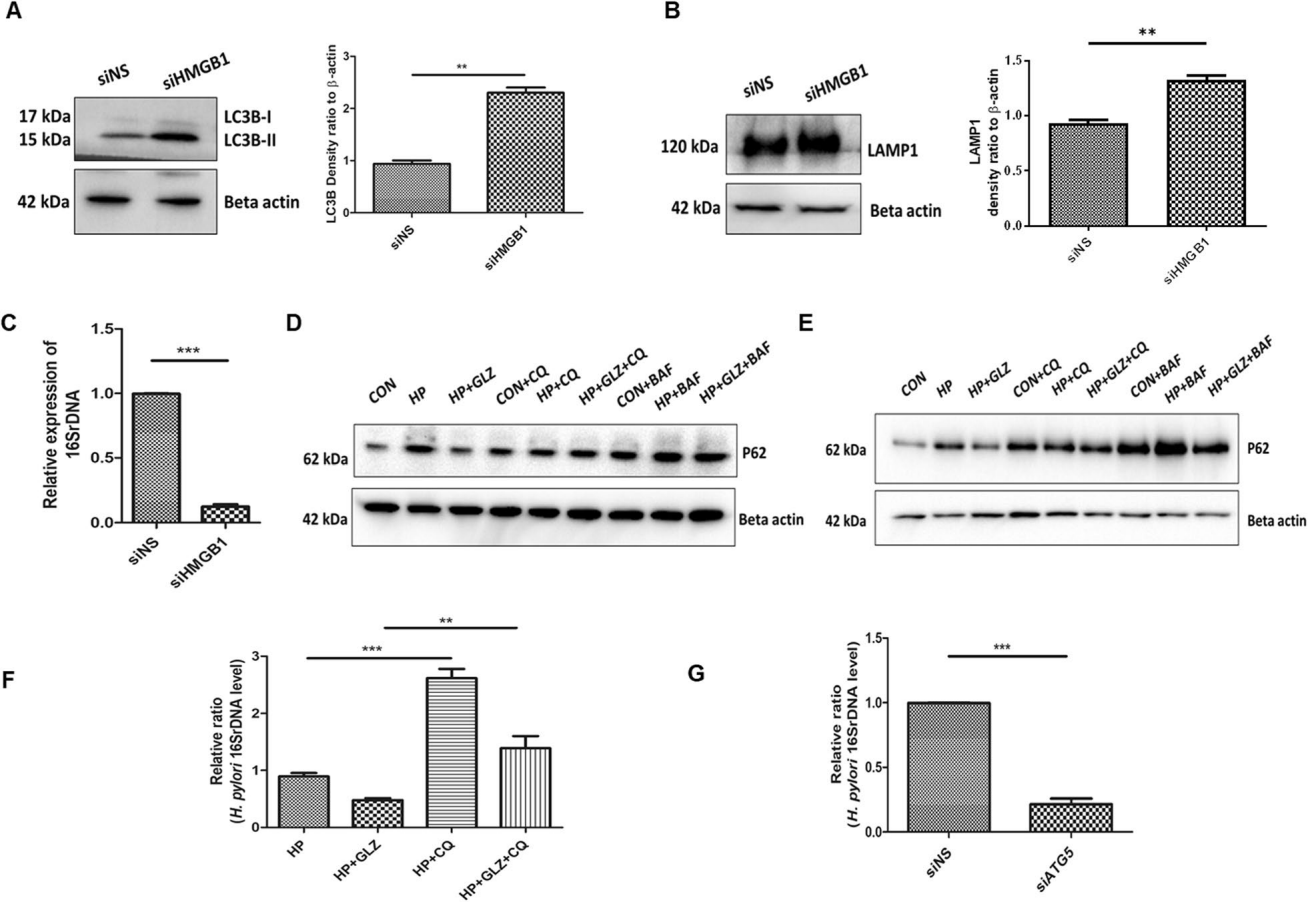
HMGB1 inhibition reduces bacterial growth while impairment of lysosomal activity induces bacterial survivability. **a–c** After transfection for 48 h, nonspecific siRNA (siNS) and HMGB1 siRNA (siHMGB1) transfected cells were further infected with *H. pylori* SS1 strain (MOI of 100) for 4 h. **a, b** Immunoblotting was performed in infected cell lysates for expression of LC3B-II and LAMP1. Beta-actin was used as a protein loading control. Densitometry analyses are represented graphically. **c** Intracellular *H. pylori* DNA (16SrDNA) was determined by RT- PCR. GAPDH was used as the internal control. Unpaired t-test was performed **(d, e)** AGS cells incubated with the *H. pylori* SS1 strain for 4 h and 18 h followed by exposure to glycyrrhizin GLZ (200 µM) and/or chloroquine CQ (50 μM) and/or bafilomycin BAF (50 nM) for 4 h and 18 h treatment respectively. Cell lysates were subjected to a western blot to determine P62 protein levels for 4 h **(d)** and 18 h **(e**). Beta-actin was used as a protein loading control. **f** AGS cells were incubated with the *H. pylori* SS1 strain for 4 h followed by exposure to glycyrrhizin GLZ (200 µM) and/or chloroquine CQ (50 μM) for 4 h. Intracellular *H. pylori* DNA (16SrDNA) was measured by RT- PCR. GAPDH was used as the internal control. **g** Cells were transfected with non-specific siRNA (siNS) and ATG5 siRNA (siATG5) & then incubated with *H. pylori* SS1 strain for 4 h, and intracellular *H. pylori* DNA was measured by RT- PCR. GAPDH was kept as an internal control. Graphs were represented as mean ± SEM (n = 3); One-way ANOVA was performed and significance was calculated; **p < 0.01, ***p < 0.001

To confirm or rule out the possible involvement of HMGB1 in *H. pylori* infection, AGS cells were transiently transfected with non-specific siRNA and HMGB1- specific siRNA followed by infection with *H. pylori* for 4 h and subjected to immunoblotting. Western blot revealed that HMGB1 is silenced in AGS cells after 48 h transfection (Additional file 1: Fig. S3A). HMGB1 knockdown elevated the level of LC3B-II by 2.2 fold change (p value: 0.0073)and LAMP1 by 1.3 fold (unpaired t-test and p value: 0.0018) significantly (Fig. 4A, B). In addition, we examined the level of intracellular *H. pylori* by RT-PCR. HMGB1 silencing attenuated intracellular *H. pylori* burden significantly by 5.9 fold (p value: 0.0001) (Fig. 4C).

Further, we confirmed the effect of glycyrrhizin on autophagic flux. One of the best targets of autophagic flux is p62. Hence, we checked the level of SQSTM1/p62, a key protein involved in autophagy. Western blotting showed that p62 is increased due to *H. pylori* infection at both 4 h (Fig. 4D) and 18 h (Fig. 4E) time points. Long-term exposure to *H. pylori*, resulted in p62 accumulation. This is evident due to the impairment of autolysosomal degradation. On the other hand, glycyrrhizin treatment induces p62 degradation at 4 h and 18 h.

To validate the effect of glycyrrhizin on autophagic flux, we treated the AGS cells with chloroquine (CQ), a potent lysosomal inhibitor, and bafilomycin (BAF), specific for inhibition of autophagosomal lysosomal fusion and acidification. P62 accumulates in both *H. pylori*-infected and uninfected cells treated with glycyrrhizin after exposure to CQ and BAF. Additionally, we analyzed the effect of CQ on glycyrrhizin-mediated *H. pylori* clearance. Due to the blocking of autophagic flux, CQ promptly elevated *H. pylori* infection by 2.9 fold and counteracted the antimicrobial action of glycyrrhizin (p value: 0.0074) (Fig. 4F).

According to previous reports, *H. pylori* can survive within non-digestive autophagosomes (Raju et al. 2012). We analyzed the effect of Atg5 knockdown (Atg5 is an autophagy marker protein required for autophagosome formation) on intracellular *H. pylori* survival. Western blot showed that Atg5 is silenced in AGS cells after 48 h transfection (Additional file 1: Fig. S3B). Consistently, bacterial clearance occurred significantly by 4.6 fold due to the silencing of Atg5 (p value: 0.0001) (Fig. 4G). Cumulatively, these data demonstrated that *H. pylori* proliferate within autophagosomes inside the host while glycyrrhizin, an inhibitor of HMGB1 promotes autophagosomal lysosomal degradation which in turn reduces intracellular *H. pylori* growth.

### Lysosomal acidification was recovered by glycyrrhizin

**Fig. 5:**
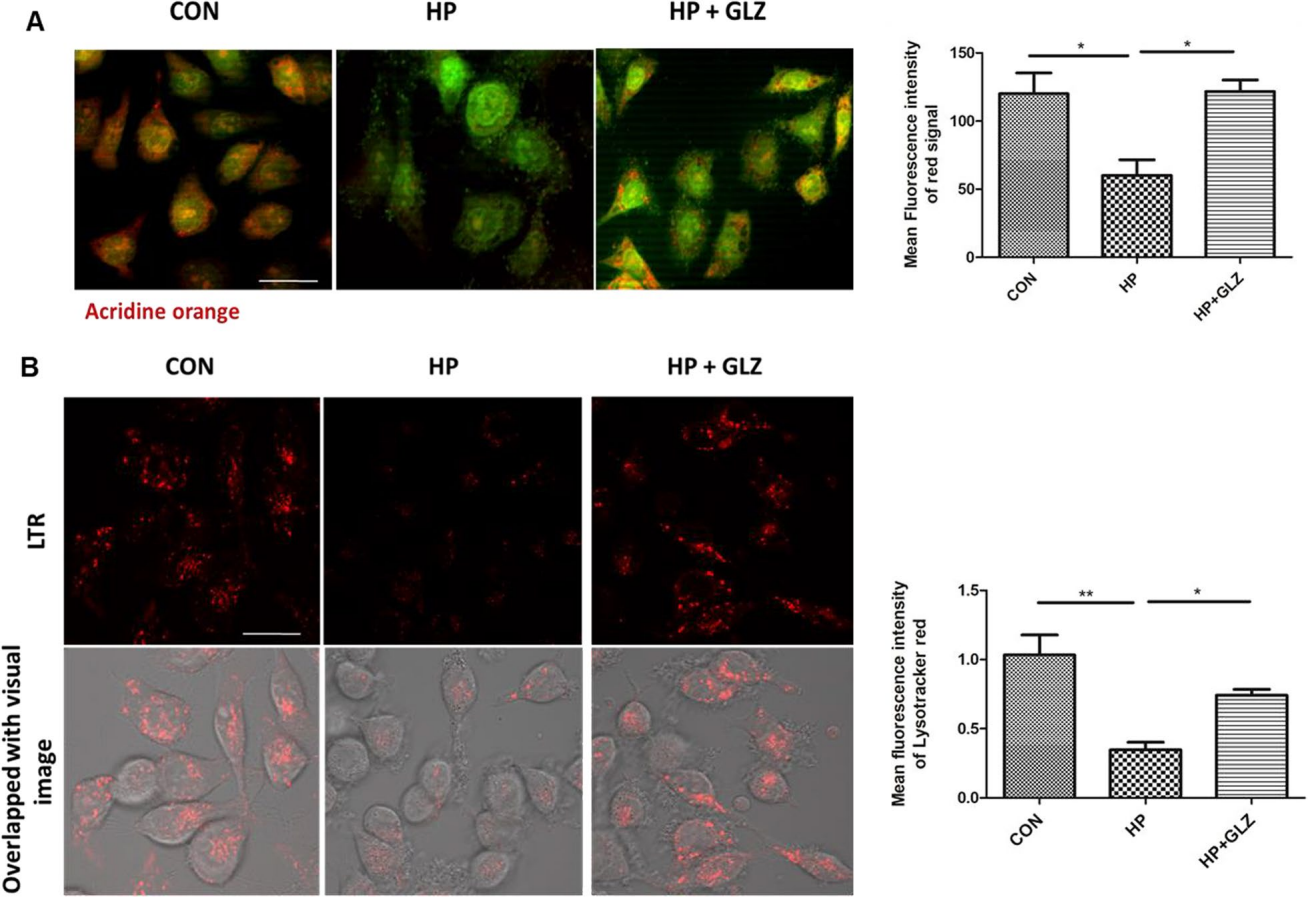
Lysosomal function is restored by glycyrrhizin. **a–b** Infection with *H. pylori* SS1 strain (MOI 100) was performed for 4 h followed by glycyrrhizin (GLZ) (200 µM) exposure for 4 h. **a** Lysosomal membrane integrity was monitored by Acridine Orange (AO) staining in a fluorescence microscope. Briefly, cells were incubated with 10 μg/ml of acridine orange (15 min) and examined. The mean fluorescence intensity of the red signal was determined and graphically represented. Scale bar: 10 μm. **b** Live cell imaging of drug-treated, infected and control cells was done with LysoTracker Red incubation (100 nM, 30 min) to label lysosomes and mean fluorescence intensity was assessed under the confocal microscope. Scale bar: 10 μm. Fold change was quantified and graphs were generated using GraphPad Prism 5 and represented as mean ± SEM (n = 3); Significance was calculated by one-way ANOVA; *p < 0.05, **p < 0.01

Further, we investigated in detail the restored lysosomal degradation capacity of glycyrrhizin. We performed Acridine Orange staining to monitor the acidic compartment of lysosomes in AGS cells. A lower red signal in *H. pylori*-infected cells was observed as compared to control because lysosomal acidification was compromised whereas glycyrrhizin treatment increased red intensity by restoring lysosomal acidification by a factor of 2.0 (p value: 0.0263) (Fig. 5A). Additionally, to evaluate lysosomal acidification, we exposed the cells to Lysotracker Red which selectively binds to vesicles that have low pH. Here, *H. pylori* infection affected lysosomal pH and reduced the fluorescent signals as compared to the control. Glycyrrhizin exposure restored the acidic pH and showed red signals in infected cells as compared to only infected cells (fold change: 2.139 and p value: 0.0495) (Fig. 5B). The data indicates that glycyrrhizin restored the disrupted lysosomal function during *H. pylori*-infection.

### Glycyrrhizin enhanced lysosomal degradation by inhibiting lysosomal membrane permeabilization (LMP)

**Fig. 6:**
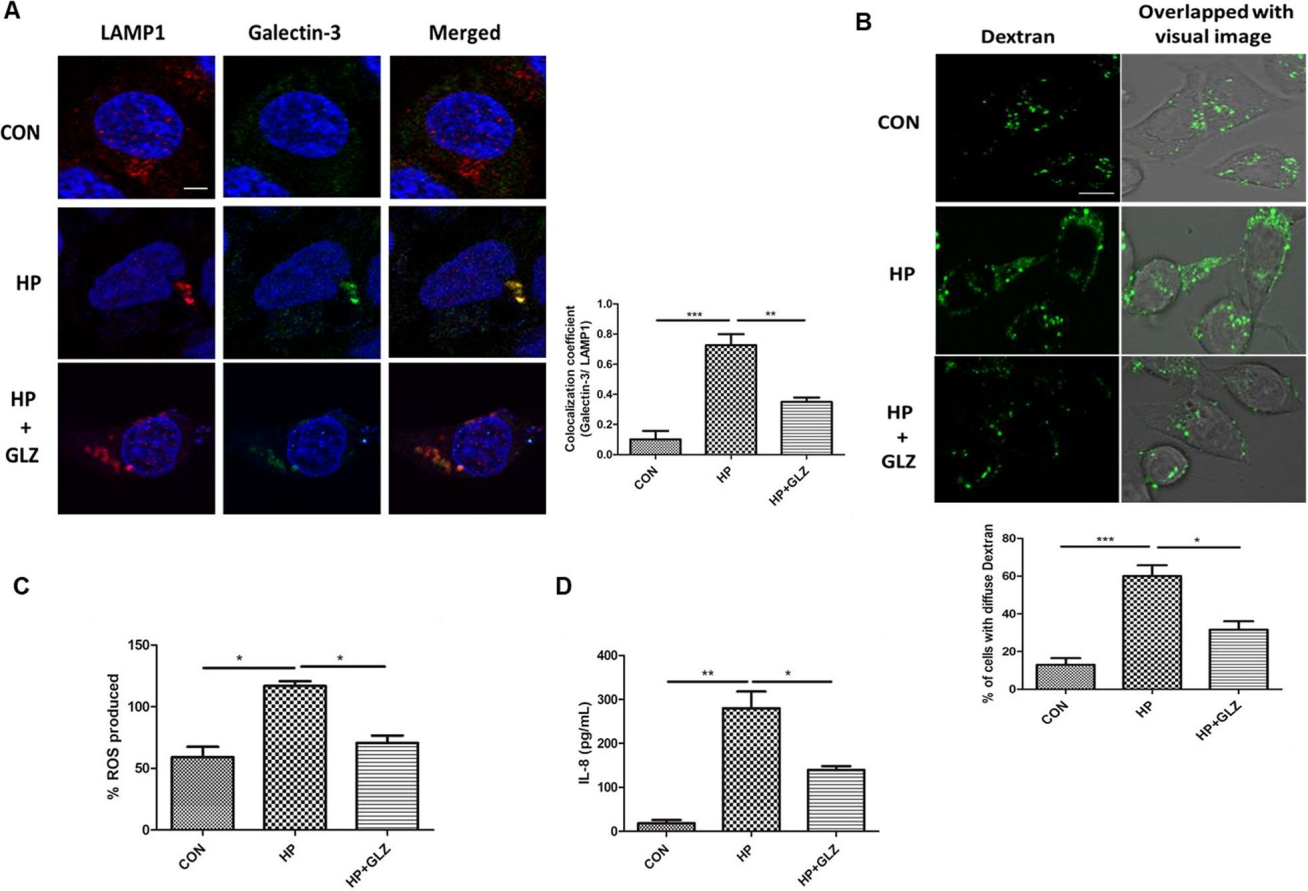
Lysosomal membrane integrity is restored by glycyrrhizin. **a–d** Infection with *H. pylori* SS1 strain (MOI 100) was performed for 4 h followed by glycyrrhizin (GLZ) (200 µM) treatment (4 h). **a** Double immunofluorescence was done with LAMP1 & Galectin-3 antibodies to confirm LMP. Cells were observed under the confocal microscope and the difference in the co-localization coefficient of each group was calculated. Scale bar: 2 μm. **b** Lysosomal destabilization was estimated using live cell imaging after staining with dextran (0.5 mg/ml) for 2 h under the confocal microscope and the number of cells with diffused dextran was counted Scale bar: 5 μm. **c** Expression of reactive oxygen species (ROS) in infected and drug-treated cells was determined by DCFDA methods for 30 min in a fluorimeter. **d** The expression of IL-8 from media collected after treatment was analyzed by ELISA in a microplate reader. Graphs were generated using GraphPad Prism 5 and represented as mean ± SEM (n = 3); Significance was calculated by one-way ANOVA; *p < 0.05, **p < 0.01, ***p < 0.001

According to previous reports, *H. pylori* reduced lysosomal degradation capacity due to lysosomal membrane permeabilization (LMP) (Bravo et al. 2019). Moreover, HMGB1 is also involved in LMP (Feng et al. 2022). Therefore, we determined whether the effect of glycyrrhizin in restoring lysosomal function is mediated by LMP. We performed double immuno-fluorescence of galectin3 and LAMP1. Colocalization of galectin3 with LAMP1 remarkably increased in *H. pylori* infection than control as galectin3 binds to lysosomal membrane glycoproteins which are exposed after LMP*.* But, glycyrrhizin treatment in infected cells reduced co-localization of galectin3 and LAMP1 probably due to inhibition of LMP (fold change: 2.07 and p value: 0.0074) (Fig. 6A). Additionally, we validated LMP by loading control, *H. pylori*-infected and drug-treated cells with Alexa Fluor conjugated dextran molecules. The redistribution of dextran was observed in infected cells (diffuse staining) indicating lysosomal efflux but not in the case of glycyrrhizin-treated infected cells. Confined punctate structures of dextran indicated exclusive lysosomal localization in drug-treated cells (Fig. 6B). Subsequently, we examined the effect of the inhibition of LMP by glycyrrhizin on ROS and inflammatory cytokines as LMP is linked to inflammation and oxidative stress. In line, glycyrrhizin-exposed cells dramatically reduced both ROS levels (fold change: 1.65 and p value: 0.0289) and IL-8 secretion (fold change: 2 and p value: 0.0456) significantly as compared to only infected cells (Fig. 6C, D). Taken together, these data indicate that glycyrrhizin improved lysosomal degradation activity and induced protective effects by inhibiting LMP.

### In in vivo mice model, glycyrrhizin induces autophagy and reduces gastric damages

**Fig. 7:**
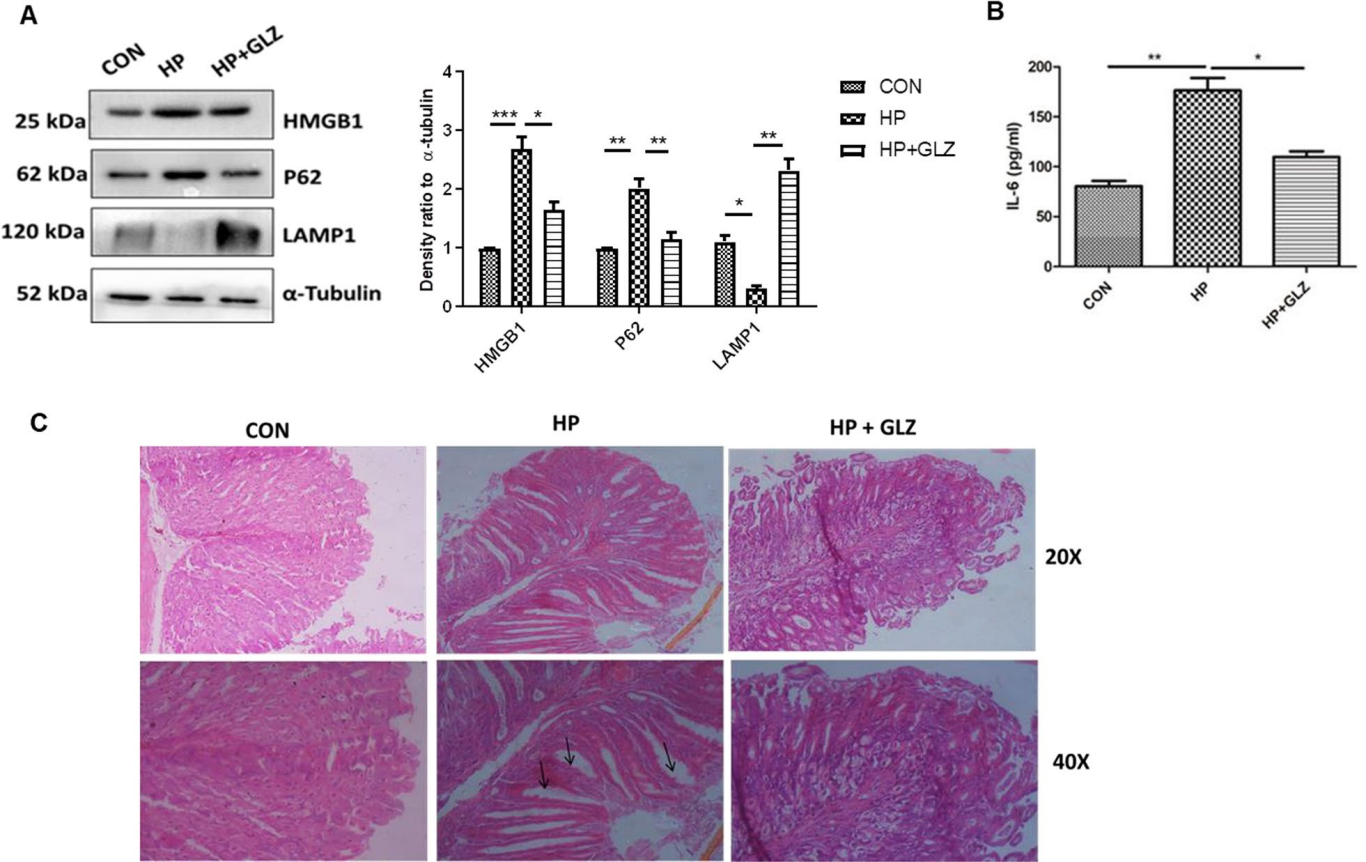
Glycyrrhizin treatment induces autophagy in *H. pylori*-infected mice and ameliorates gastric tissue damage **(a–c)** C57BL/6 mice (n = 5 per group) were treated with antibiotics every 7 days. Then after 7 days incubation period, mice were infected with the *H. pylori* SS1 strain thrice a week on alternate days. Mice were incubated for 14 days and then administered with or without glycyrrhizin GLZ (10 mg /kg body weight), every day for 4 weeks. After treatment mice were sacrificed, and gastric tissues and serum were collected. **a** Immunoblot showing the expression of HMGB1 and autophagy proteins (HMGB1, P62, LAMP1) of mouse gastric tissues. α-tubulin was used as a protein loading control. Densitometry analyses are represented graphically. **b** The expression of IL-6 was determined by ELISA in a microplate reader. **c** Histology images of Control (CON*), H. pylori* (HP) infected, and *H. pylori*-infected plus glycyrrhizin treated (HP + GLZ) gastric tissues at 20X and 40X respectively representing the inflammatory changes. Black arrows (↑) indicate gastric tissue damages. Graphs were represented as mean ± SEM (n = 3); Significance was determined by one-way ANOVA; *p < 0.05, **p < 0.01, ***p < 0.001

Eventually, we validated the activity of glycyrrhizin in in vivo mice model. We have infected mice with the *H. pylori* SSI strain. After infection, mice were treated with glycyrrhizin for 30 days at a 10 mg/kg body weight dose (Additional file 1: Fig. S4A). The effective dose of glycyrrhizin was determined for *H. pylori*-infected mice from previous data on glycyrrhizin (Lv et al. 2020; Fu et al. 2014). We performed western blots with control, infected, and glycyrrhizin-treated-infected gastric tissues. Glycyrrhizin reduced the level of HMGB1 significantly by 1.64 fold (p value: 0.0262) and induced p62 degradation by 1.73 fold (p value: 0.0010) which is a marker of autophagosomal lysosomal degradation. It also strongly augmented LAMP1 expression by 7.55 fold (p value: 0.003) (Fig. 7A). Furthermore, we checked the effect of glycyrrhizin on IL-6 levels in the serum collected from treated mice. Glycyrrhizin significantly reduced IL-6 expression by a factor of 1.6 (p value: 0.0086) (Fig. 7B). To further assess the anti-*H. pylori* effect of glycyrrhizin, we examined gastric tissues for changes in morphology. There were changes in the gastric histopathology when compared with a control group for *H. pylori*-infected gastric tissues (Fig. 7C). *H. pylori-*induced inflammation and inflammatory cell infiltration in gastric tissues caused epithelial cell damage whereas glycyrrhizin reduced inflammation and repaired tissue damage. Hence, collectively these data revealed that glycyrrhizin induced autophagy and consequently decreased inflammation and gastric tissue damage in in vivo mice model.

## Discussion

Current evidence suggests that autophagy plays a major role to protect the host from bacterial pathogens (Giraud-Gatineau et al. 2020; Kim et al. 2012). But the pathogens have their mechanisms to subvert autophagy and persistently invade the host and promote intracellular survival. *Salmonella*, *Shigella,* and *Mycobacterium* are known to avoid autophagy (Xie et al. 2020; Ogawa et al. 2005; Basak et al. 2022; Padhi et al. 2019). However, there are reports which showed that *H. pylori* induce autophagy at the beginning but gradually it inhibits autophagy (Yang et al. 2018; Tang et al. 2012). The mechanisms behind initial activation and subsequent impairment involve a complex interplay between host and bacterial factors. *H. pylori* secretes virulent factors like CagA and VacA (Raju et al. 2012). Both these factors contribute to pathogenesis. Although previous literature suggests that VacA induces autophagy but later it has been reported by Raju et al. that prolonged exposure to VacA inhibits autophagy (Raju et al. 2012; Terebiznik et al. 2009). Recent studies have also revealed that autophagy is down-regulated in *cagA* + strains as compared to *cagA* mutant strains. This inhibition of autophagy is accompanied by the accumulation of p62 and decreased LAMP1 expression (Li et al. 2017). Henceforth, in the current study, we have used the *H. pylori* SS1 strain which is *cagA* positive but expresses non-functional *vacA* and this strain is also capable of mice infection.

Here, we investigated the effect of HMGB1 inhibition during *H. pylori* infection. HMGB1 is reported to be overexpressed in *H. pylori*-infected gastric cells (Lin et al. 2016). Recent reports suggested that HMGB1 causes impairment of autophagy by inducing lysosomal membrane permeabilization (LMP) in diabetic retinopathy (Feng et al. 2022). Hence, we have targeted HMGB1 for treating *H. pylori* infection. Here, glycyrrhizin, an inhibitor of HMGB1 decreased the intracellular *H. pylori* burden in gastric cancer cells. To find out the details behind bacterial clearance, we observed that glycyrrhizin induces autophagy in gastric cells. This is consistent with previous studies of glycyrrhizin in myoblast cells (Lv et al. 2020). Glycyrrhizin treatment attenuated *H. pylori* infection and also induced the expression of autophagy marker proteins. Moreover, glycyrrhizin showed co-localization of both LC3B and LAMP1 in *H. pylori*-infected gastric cancer cells. We also observed that LAMP1 expression has increased due to glycyrrhizin treatment. This indicates autolysosome formation as LAMP1 expression is necessary for autophagosomal maturation (Tsugawa et al. 2019). Keeping in mind that antibiotic resistance of *H. pylori* is a major problem (Gene et al. 2003; Huang et al. 2017), glycyrrhizin was tested for its ability to eradicate the growth of resistant *H. pylori* strain in in vitro conditions. Glycyrrhizin successfully showed clearance of antibiotic-resistant *H. pylori.* In addition, transient knock-down of HMGB1 resulted in parallel to glycyrrhizin treatment, hence the probable mechanism behind the induction of autophagy by glycyrrhizin is due to its inherent anti- HMGB1 property. Bacterial clearance by autophagy induction is a general mechanism as recent reports revealed that autophagy inducers like vitamin D and statin controlled *H. pylori* infection (Hu et al. 2019; Liao et al. 2017). Further, we determined the effect of glycyrrhizin in the later stages of autophagy. Consistent with the results of initial autophagy induction, our results demonstrated that glycyrrhizin treatment augments lysosomal degradation resulting in reduced bacterial burden. It is reported that *H. pylori* induce p62 accumulation which is a characteristic feature of inhibition of autophagic flux. In contrast, glycyrrhizin treatment augmented p62 degradation to improve the autophagic flux. Subsequently, p62 accumulation was observed due to the inhibition of autophagosomal maturation and flux by chloroquine and bafilomycin**.** Glycyrrhizin further is unable to inhibit *H. pylori* growth due to CQ treatment indicating the involvement of autophagic flux during infection **(**Hu et al. 2019). To gain a deeper insight into autophagic activity by glycyrrhizin we searched for the probable mechanisms. Previous studies suggested that *H. pylori* survived in undigested autophagosomes (Raju et al. 2012). Consistently*,* we proved that Atg5 knockdown inhibited *H. pylori* growth as Atg5 is responsible for autophagosome formation*.* Furthermore, the accumulation of undigested autophagosomes results in lysosomal membrane permeabilization (LMP) (Feng et al. 2022). HMGB1 is associated with LMP causing defective lysosomal activity and *H. pylori* is also reported to have impairment of lysosomal acidification by inducing LMP (Bravo et al. 2019; Feng et al. 2022). In this study, inhibition of HMGB1 by glycyrrhizin rescued LMP in *H. pylori*-infected cells and further restored the degradative capacity of autophagy proving that LMP is responsible for the inhibition of autophagosome degradation. Moreover, we observed that lysosomal pH has been restored by glycyrrhizin using Lysotracker Red staining as acidic pH is extremely important for the proper digestive action of lysosomes. Further, we examined Alexa Fluor conjugated dextran activity to inhibition of LMP by glycyrrhizin. Here, also we found that glycyrrhizin exposure reduced LMP by inducing consistent puncta formation of dextran granules indicating intact lysosomes whereas in infected cells diffuse staining is observed due to lysosomal efflux. Additionally, both HMGB1 and *H. pylori* infection are responsible for LMP (Bravo et al. 2019; Feng et al. 2022). Thus it has been proved that glycyrrhizin induces autophagy and lysosomal degradation by reducing LMP. Moreover, LMP is linked to the activation of ROS and inflammation (Kavčič et al. 2017). We evaluated the effect on ROS generation and cytokine expression. ROS level and inflammatory cytokine expression are commonly enhanced during infection (Hardbower et al. 2013). Consistently, the results showed that glycyrrhizin inhibited ROS production and inflammatory cytokine level in AGS cells. Therefore, our findings confirmed that inhibiting HMGB1 by glycyrrhizin induced autophagosomal maturation and lysosomal degradation in *H. pylori*-infected gastric cells by rescuing them from LMP.

Further, we verified our findings in in vivo mice model. In line, our results showed inhibition of HMGB1 expression and induction of autophagy by glycyrrhizin in gastric tissues. Inflammation is a major problem during *H. pylori* infection. Inflammation induces gastric damage and causes further complications. Here, glycyrrhizin induces autophagy accompanied by a reduction in inflammation. Furthermore, glycyrrhizin is able to repair gastric tissue damages.

## Conclusion

**Fig. 8:**
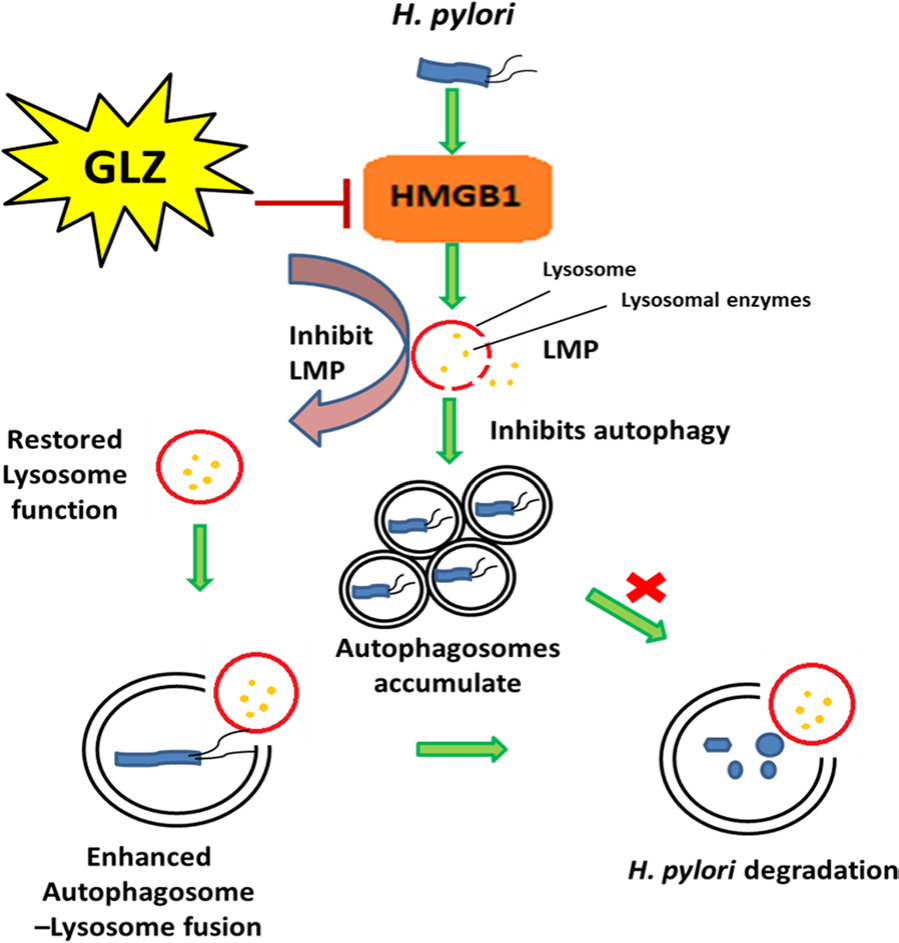
Schematic diagram represents the mechanism that inhibiting HMGB1 induces autophagy through lysosomal membrane permeabilization(LMP). Glycyrrhizin inhibiting the HMGB1 expression induces autophagy by restoration of lysosomal membrane integrity. Recovered lysosomal membrane reduces LMP along with enhanced autolysosome formation which ultimately degrades the intracellular *H. pylori* from its gastric niche

Our results demonstrated for the first time that induction of autophagy by inhibiting HMGB1 can reduce *H. pylori* infection in both in vitro and in vivo conditions. In addition, our data revealed that restoring the degradative capacity of autophagy by inhibiting HMGB1- induced LMP resulted in the inhibition of *H. pylori* pathogenesis (Fig. 8). As both the host and pathogen play critical roles in disease progression, induction of autophagy and lysosomal degradation further affects downstream responses like inflammation and ROS generation. Hence, in the future glycyrrhizin might be used as a potent inducer of autophagy that reduces *H. pylori* infection to inhibit the progression of gastric disorders. The mechanism of antibacterial action of glycyrrhizin would provide novel strategies and targets to address the problem of antimicrobial resistance. Glycyrrhizin could also be used in synergistic composition with other drugs for *H. pylori* infection as standard *H. pylori* treatment requires triple therapy.

## Supplementary Information

**Additional file 1: Figure S1. **MTT assay of glycyrrhizin for different doses. (a) AGS cells were treated with glycyrrhizin (50-200µM) or DMSO for 24 h. MTT assay was performed to measure the % viability level. Densitometric analyses are graphically represented. Graphs generated using GraphPad Prism 5 were represented as mean±SEM (n=3); One-way ANOVA was performed and significance was calculated. ns=nonsignificant. **Figure S2.** Standard agar dilution method for determination of *Helicobacter pylori* viability. Briefly,  serially diluted bacterial suspension of OD at 600nm 0.1 were spotted on BHIA medium containing glycyrrhizin of 200µM concentration along with control where no glycyrrhizin was added and incubated them in the microaerophilic condition for 3-4 days. *H. pylori* viability was determined by counting the number of bacterial colonies (CFU/mL) in the BHIA medium. Graphs generated using GraphPad Prism 5 were represented as mean±SEM (n=3); One-way ANOVA was performed and significance was calculated. ns=nonsignificant. **Figure S3.** Transfection of siHMGB1, siATG5 and nonspecific siRNA in AGS cells. (a) Cells were transfected with non-specific siRNA (siNS) and HMGB1 siRNA for 48h. Immunoblotting was performed for quantification of HMGB1 inhibition. (b) Cells were transfected with non-specific siRNA (siNS) and ATG5 siRNA (siATG5) for 48h. Immunoblotting was performed for quantification of ATG5 inhibition. Beta-actin was used as a loading control. **Figure S4.** Glycyrrhizin treatment in *H. pylori*-infected mice. C57BL/6 mice (n = 5 per group) were treated with antibiotics every 7 days. Then after 7 days incubation period, mice were infected with the *H. pylori* SS1 strain thrice a week on alternate days. Mice were incubated for 14 days and then administered with or without GLZ (10 mg /kg body weight), every day for 4 weeks. At the end of treatment, mice were sacrificed and gastric tissues and serum were collected. (b) Immunoblot showing the expression of autophagy proteins LC3B-II of mouse gastric tissues. α-tubulin was used as a protein loading control.

## Data Availability

All required data included in text and supplementary. Any further any formation required is available with the corresponding author.
