# Supplementary material for: Glycyrrhizin, an inhibitor of HMGB1 induces autolysosomal degradation function and inhibits Helicobacter pylori infection
Source: Mol Med. 2023 Apr 10;29:51. doi: 10.1186/s10020-023-00641-6 (PMC10088177; doi:10.1186/s10020-023-00641-6)
Supplement: Supplementary file 1 — Additional file 1: Figure S1. MTT assay of glycyrrhizin for different doses. (a) AGS cells were treated with glycyrrhizin (50-200µM) or DMSO for 24 h. MTT assay was performed to measure the % viability level. Densitometric analyses are graphically represented. Graphs generated using GraphPad Prism 5 were represented as mean±SEM (n=3); One-way ANOVA was performed and significance was calculated. ns=nonsignificant. Figure S2. Standard agar dilution method for determination of Helicobacter pylori viability. Briefly, serially diluted bacterial suspension of OD at 600nm 0.1 were spotted on BHIA medium containing glycyrrhizin of 200µM concentration along with control where no glycyrrhizin was added and incubated them in the microaerophilic condition for 3-4 days. H. pylori viability was determined by counting the number of bacterial colonies (CFU/mL) in the BHIA medium. Graphs generated using GraphPad Prism 5 were represented as mean±SEM (n=3); One-way ANOVA was performed and significance was calculated. ns=nonsignificant. Figure S3. Transfection of siHMGB1, siATG5 and nonspecific siRNA in AGS cells. (a) Cells were transfected with non-specific siRNA (siNS) and HMGB1 siRNA for 48h. Immunoblotting was performed for quantification of HMGB1 inhibition. (b) Cells were transfected with non-specific siRNA (siNS) and ATG5 siRNA (siATG5) for 48h. Immunoblotting was performed for quantification of ATG5 inhibition. Beta-actin was used as a loading control. Figure S4. Glycyrrhizin treatment in H. pylori-infected mice. C57BL/6 mice (n = 5 per group) were treated with antibiotics every 7 days. Then after 7 days incubation period, mice were infected with the H. pylori SS1 strain thrice a week on alternate days. Mice were incubated for 14 days and then administered with or without GLZ (10 mg /kg body weight), every day for 4 weeks. At the end of treatment, mice were sacrificed and gastric tissues and serum were collected. (b) Immunoblot showing the expression of autophagy proteins LC3B- [file 10020_2023_641_MOESM1_ESM.docx]

**Supplementary Figures**

**Figure S1**


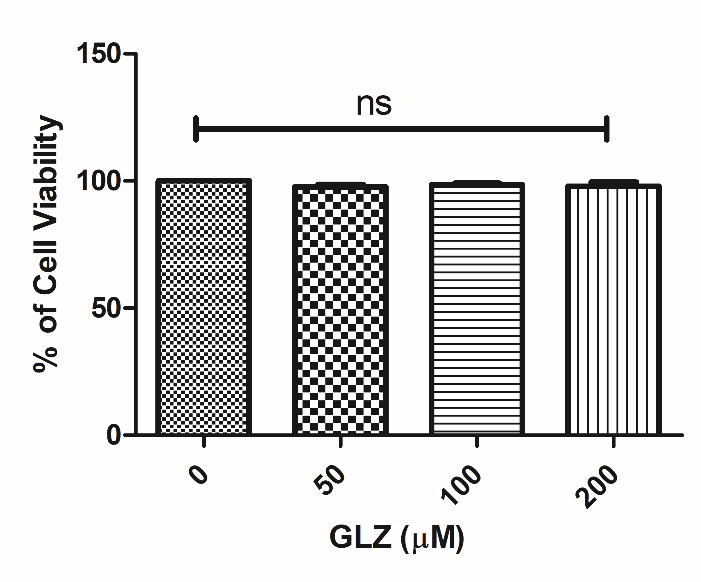


**A**

**Fig. S1** MTT assay of glycyrrhizin for different doses. (a) AGS cells were treated with glycyrrhizin (50-200µM) or DMSO for 24 h. MTT assay was performed to measure the % viability level. Densitometric analyses are graphically represented. Graphs generated using GraphPad Prism 5 were represented as mean±SEM (n=3); One-way ANOVA was performed and significance was calculated. ns=nonsignificant

**Figure S2**


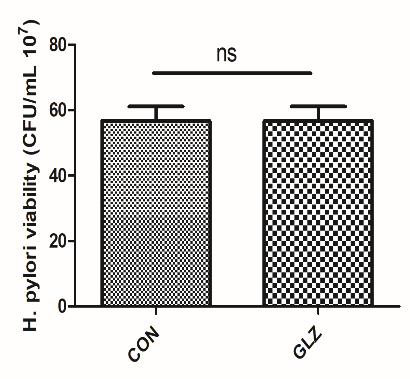


**A**

**Fig. S2** Standard agar dilution method for determination of *Helicobacter pylori* viability. Briefly, serially diluted bacterial suspension of OD at 600nm 0.1 were spotted on BHIA medium containing glycyrrhizin of 200µM concentration along with control where no glycyrrhizin was added and incubated them in the microaerophilic condition for 3-4 days. *H. pylori* viability was determined by counting the number of bacterial colonies (CFU/mL) in the BHIA medium. Graphs generated using GraphPad Prism 5 were represented as mean±SEM (n=3); One-way ANOVA was performed and significance was calculated. ns=nonsignificant

**Figure S3**

**B**

**A**

**
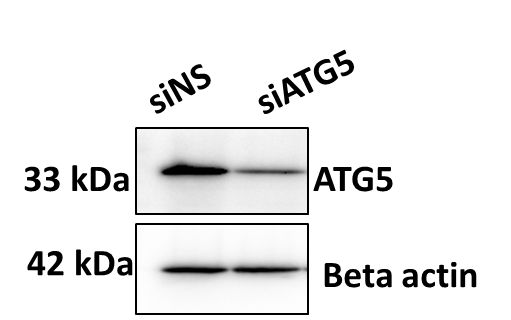

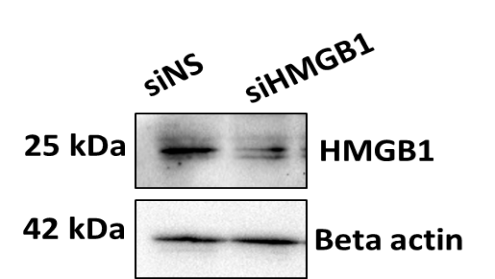
**

**Fig. S3 Figure S3 Transfection of siHMGB1, siATG5 and nonspecific siRNA in AGS cells.** (a) Cells were transfected with non-specific siRNA (siNS) and HMGB1 siRNA for 48h. Immunoblotting was performed for quantification of HMGB1 inhibition. (b) Cells were transfected with non-specific siRNA (siNS) and ATG5 siRNA (siATG5) for 48h. Immunoblotting was performed for quantification of ATG5 inhibition. Beta-actin was used as a loading control.

**Figure S3**

**A**

**Figure S4**


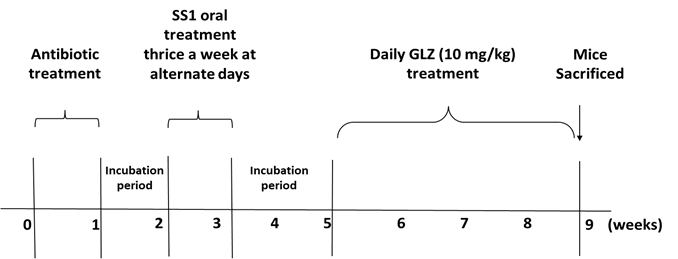


**Fig. S4** Glycyrrhizin treatment in *H. pylori*-infected mice. C57BL/6 mice (n = 5 per group) were treated with antibiotics every 7 days. Then after 7 days incubation period, mice were infected with the *H. pylori* SS1 strain thrice a week on alternate days. Mice were incubated for 14 days and then administered with or without GLZ (10 mg /kg body weight), every day for 4 weeks. At the end of treatment, mice were sacrificed and gastric tissues and serum were collected. (b) Immunoblot showing the expression of autophagy proteins LC3IIB of mouse gastric tissues. α-tubulin was used as a protein loading control.
